# Altered Functional Connectivity Within and Between Salience and Sensorimotor Networks in Patients With Functional Constipation

**DOI:** 10.3389/fnins.2021.628880

**Published:** 2021-03-11

**Authors:** Shijun Duan, Lei Liu, Guanya Li, Jia Wang, Yang Hu, Wenchao Zhang, Zongxin Tan, Zhenzhen Jia, Lei Zhang, Karen M. von Deneen, Yi Zhang, Yongzhan Nie, Guangbin Cui

**Affiliations:** ^1^Department of Radiology, Tangdu Hospital, Air Force Medical University, Xi’an, China; ^2^Center for Brain Imaging, School of Life Sciences and Technology, Xidian University, Xi’an, China; ^3^State Key Laboratory of Cancer Biology, National Clinical Research Center for Digestive Diseases and Xijing Hospital of Digestive Diseases, Air Force Medical University, Xi’an, China

**Keywords:** functional constipation, anxiety/depression, resting-state fMRI, brain connectome, graph-theory approach, modularity

## Abstract

Functional constipation (FCon) is a common functional gastrointestinal disorder. A considerable portion of patients with FCon is associated with anxiety/depressive status (FCAD). Previous neuroimaging studies mainly focused on patients with FCon without distinguishing FCAD from FCon patients without anxiety/depressive status (FCNAD). Differences in brain functions between these two subtypes remain unclear. Thus, we employed resting-state functional magnetic resonance imaging (RS-fMRI) and graph theory method to investigate differences in brain network connectivity and topology in 41 FCAD, 42 FCNAD, and 43 age- and gender-matched healthy controls (HCs). FCAD/FCNAD showed significantly lower normalized clustering coefficient and small-world-ness. Both groups showed altered nodal degree/efficiency mainly in the rostral anterior cingulate cortex (rACC), precentral gyrus (PreCen), supplementary motor area (SMA), and thalamus. In the FCAD group, nodal degree in the SMA was negatively correlated with difficulty of defecation, and abdominal pain was positively correlated with nodal degree/efficiency in the rACC, which had a lower within-module nodal degree. The salience network (SN) exhibited higher functional connectivity (FC) with the sensorimotor network (SMN) in FCAD/FCNAD, and FC between these two networks was negatively correlated with anxiety ratings in FCAD group. Additionally, FC of anterior insula (aINS)–rACC was only correlated with constipation symptom (i.e., abdominal pain) in the FCNAD group. In the FCAD group, FCs of dorsomedial prefrontal cortex–rACC, PreCen–aINS showed correlations with both constipation symptom (i.e., difficulty of defecation) and depressive status. These findings indicate the differences in FC of the SN–SMN between FCAD and FCNAD and provide neuroimaging evidence based on brain function, which portrays important clues for improving new treatment strategies.

## Introduction

As a typical functional gastrointestinal disorder (De Giorgio et al., 2015; Koppen et al., 2015), functional constipation (FCon) is featured by infrequent bowel movement, painful defecation, hard and/or large stools, and sensation of incomplete evacuation and is often accompanied by abdominal distension/pain (Alame and Bahna, 2012; Koppen et al., 2015; Zhu et al., 2016; Jin et al., 2019). The prevalence of FCon ranged from 0.7 to 79% (median 16%) in the worldwide general population (Mugie et al., 2011), and a considerable proportion of patients with FCon has anxiety and depression (Cheng et al., 2003; Hosseinzadeh et al., 2011). These FCon symptoms in addition to altered psychological states have a severe impact on patients’ health-related quality of life and brain (Glia and Lindberg, 1997; Bongers et al., 2009).

Magnetic resonance imaging (MRI) is increasingly being used to evaluate changes in brain regions and neural circuitries involved in processing visceral stimuli in patients with FCon. One resting-state functional MRI (RS-fMRI) study in patients with FCon (14 patients in total) revealed alterations in brain regions implicated in emotional process modulation [i.e., dorsal anterior cingulate cortex (dACC), insula (INS), orbital frontal cortex (OFC), and hippocampal gyrus (HIPP)], somatic/motor control [i.e., precentral gyrus (PreCen) and supplementary motor area (SMA)] (Zhu et al., 2016). Another RS-fMRI study investigated sex-related differences in brain activity and functional connectivity (FC) in 51 patients with FCon who showed lower baseline brain activities (i.e., INS, OFC, thalamus, PreCen) in the female FCon group (34 females) compared to males and a negative association between INS/OFC and constipation symptoms (Jin et al., 2019). One newly published article examined the topological organization of an intrinsic brain functional network in 42 patients with FCon and demonstrated that the thalamus-related network exhibited altered connections with the limbic network including the amygdala and HIPP, depicting that FCon was associated with dysfunction in the thalamocortical network (Liu et al., 2020). In addition, structural MRI has been employed to assess the structural alterations that might be related to brain functional changes. One recently published paper that investigated 29 patients with FCon reported decreased cortical thickness in the dACC, OFC, posterior cingulate cortex (PCC)/precuneus, and SMA in that group (Hu et al., 2020). Another study employed diffusion tensor imaging with probabilistic tractography reflecting that FCon is associated with alterations in structural connectivity between the thalamus and limbic/parietal cortex by investigating 29 patients with FCon (Zhang et al., 2020). These findings revealed brain functional and structural changes by comparing FCon patients with healthy controls (HCs).

However, growing evidence has shown a tight association between FCon and mental status (Dykes et al., 2001; Chan et al., 2005; Dong et al., 2013; Waters et al., 2013). It has been reported that constipated patients with slow bowel transit were associated with more psychosocial distress (Dykes et al., 2001), while Chan et al. (2005) revealed that defective coping mechanisms used by patients with FCon led to internalization of stress. Patients with FCon associated with anxiety/depressive status (FCAD) reported that their recurrent symptoms of constipation were difficult to alleviate (Chan et al., 2005), especially in children with anxiety disorders (Waters et al., 2013). These findings suggested that physiological changes to the gastrointestinal system may affect patients’ emotional status and psychological factors, which can influence visceral function (Waters et al., 2013).

Physicians noticed the above phenomenon and prescribed constipation relief and antipsychotic drugs to treat constipation, but the efficacy was limited (Canbulat and Demirgoz, 2017; Janssen et al., 2018). This fact suggested that these medications might not be effective for patients with FCAD. Those aforementioned brain imaging studies mainly focused on brain alterations in patients with FCon as compared to HCs, without paying attention to the impact of psychological factors on brain functions. As far as we know, there is only one study that has been performed to examine the alterations in brain activity in FCAD (Li et al., 2021). In this study, Li et al. (2021) reported that both FCAD (37 patients) and patients without anxiety/depressive status (FCNAD, 28 patients) showed decreased brain activity than HC in regions implicated in emotional–arousal (perigenual ACC, DMPFC) and self-referential processing (precuneus); they had enhanced PreCen–thalamus connectivity and attenuated precuneus–thalamus connectivity. Further analysis revealed that FCAD also had decreased activity than FCNAD/HC in the OFC and thalamus and showed increased OFC–hippocampus connectivity. These findings only revealed alterations in local brain region activities/FCs instead of a network perspective. Thus, it is necessary to unveil the differences in brain functions between FCAD and FCNAD from a system-level perspective; once these issues have been addressed, diagnosis and treatment for FCAD could be more effective.

Previous neuroimaging studies in patients with irritable bowel syndrome, a typical subtype of functional gastrointestinal disorder, identified altered FC among regions within a SN (Icenhour et al., 2017) in patients with irritable bowel syndrome associated with higher anxiety/depressive status. These studies highlighted the important role of the SN, which was implicated in the detection and integration of emotional and sensory stimuli in patients with functional gastrointestinal disorder (Menon, 2015). Dysfunctions of the sensorimotor network (SMN) were frequently reported in patients with functional gastrointestinal disorder (Kano et al., 2018; Nan et al., 2020). No study has been conducted to investigate the changes in brain functions in FCAD and FCNAD from a network perspective. These findings shed light on the possibility of examining brain alterations within and between resting-state networks in patients with FCon, particularly the differences between FCAD and FCNAD, in order to understand the role of psychological factors in the development of FCon.

Modules (or community structures) are topologically defined as subsets of highly interconnected nodes, which are relatively sparsely connected to nodes in other modules (Meunier et al., 2010). Brain module detection is based on graph theoretical analysis, which is a powerful tool to investigate interaction patterns among brain modules (Bullmore and Sporns, 2009). Alterations in the modules of the brain have been observed in several neuropsychiatric conditions, including schizophrenia (Stam, 2014), depression (Peng et al., 2014), FCon (Liu et al., 2020), and chronic pain (Balenzuela et al., 2010). Assessment of the brain modular organization can provide a key to understanding the relation between aberrant connectivity and brain disease (Nicolini et al., 2017). Therefore, modular organization is helpful for further understanding the central pathogenesis of FCon psychological subtypes by investigating altered FC within and between resting-state networks in patients with FCon associated with different mental (anxiety/depression) statuses.

We employed RS-fMRI and graph theory method to investigate the topological organization of the intrinsic functional brain network among 41 FCAD, 42 FCNAD, and 43 HCs. We hypothesized that there were differences in FC within and between resting-state networks between FCAD and FCNAD.

## Materials and Methods

### Participants

Patients with FCon were recruited from Xijing Hospital of digestive diseases affiliated with the Air Force Military Medical University in Xi’an, China. Diagnosis of FCon was made by a gastroenterologist experienced in the diagnosis of functional gastrointestinal disorder using Rome IV criteria (Drossman, 2016). Patients with FCon who displayed all types of predominant bowel habits (slow transit constipation, functional defecatory disorders, and combination of the two types) were recruited to participate in this study. Patients with redundant sigmoid colon/congenital giant colon/pelvic floor muscle relaxation, constipation after childbirth, neurological/mental/medical disorders requiring immediate treatment, and current medications that could affect the central nervous system were excluded from the experiment. Eighty-three remaining patients with FCon completed the MRI scans. Before the experiment, each participant had already read and signed an informed consent form. HCs were recruited from the local community and consisted of 43 age- and gender-matched subjects (Table 1). A series of self-administered questionnaires was given to all participants to complete, including difficulty of defecation, sensation of incomplete evacuation, and abdominal distension/pain. They were also asked to complete the ZUNG self-rating depressive scale (SDS) (Zung, 1965), ZUNG self-rating anxiety scale (SAS) (Zung, 1971), and the State-Trait Anxiety Inventory (STAI) (Shek, 1993) to assess their severity of depression/anxiety. The experimental protocol was approved by the institutional review board of Xijing Hospital and was registered in the Chinese Clinical Trial Registry Center as ChiCTR-OOB-15006347^1^. The experiments were conducted in accordance with the Declaration of Helsinki.

**TABLE 1:** Demographic and clinical information of patients with FCAD, FCNAD, and HC subjects.

		HC (*N* = 43)	FCon (*N* = 83) (Mean ± SE)		One-way ANOVA		*Post hoc*		
		(Mean ± SE)	FCNAD (*N* = 42)	FCAD (*N* = 41)	*F*	*p*	*a*	*b*	*c*
Age (years)		38.0698 ± 2.0942	42.7619 ± 2.0774	38.6098 ± 1.8044	1.6503	0.1962	0.0980	0.8491	0.1473
Gender		18M/25F	16M/26F	10M/31F	3.0981	0.2124	0.4473	0.0709	0.1336
Depression (SDS)		37.3953 ± 1.1780	44.7857 ± 1.0710	69.0244 ± 1.4673	167.1536	<0.001	<0.001	<0.001	<0.001
Anxiety (SAS)		33.4884 ± 1.0462	41.3810 ± 1.0421	63.7073 ± 1.5801	158.6270	<0.001	<0.001	<0.001	<0.001
STAI	State anxiety (SAI)	28.3256 ± 1.2325	33.6667 ± 1.5406	57.3415 ± 1.5176	115.0761	<0.001	0.009	<0.001	<0.001
	Trait anxiety (TAI)	30.0465 ± 1.1489	36.3571 ± 1.4301	55.2439 ± 1.2854	102.2930	<0.001	0.001	<0.001	<0.001
Difficulty of defecation (0–100)		N/A	67.9762 ± 3.1373	71.6585 ± 3.6181	N/A	N/A	N/A	N/A	0.3445
Sensation of incomplete evacuation (0–100)		N/A	63.7619 ± 3.9184	65.5366 ± 4.8007	N/A	N/A	N/A	N/A	0.7242
Abdominal distension (0–100)		N/A	44.6429 ± 4.6566	63.5122 ± 4.6608	N/A	N/A	N/A	N/A	<0.001
Abdominal pain (0–100)		N/A	25.7857 ± 3.6878	34.9024 ± 4.6353	N/A	N/A	N/A	N/A	0.0596

*a: cross group comparison between functional constipation and healthy controls (FCNAD vs. HC) to examine the effect of constipation. b: cross group comparison between (FCAD vs. HC) to investigate the effects of constipation and anxiety/depressive status. c: cross group comparison between (FCAD vs. FCNAD) to examine the effect of anxiety/depressive status. FCNAD, functional constipation without anxiety/depressive status; FCAD, functional constipation associated with anxiety/depressive status; HC, healthy control; SE, standard error; SDS, ZUNG Self-rating Depressive Scale; SAS, ZUNG Self-rating Anxiety Scale.*

### Subtypes of Functional Constipation Classification

In order to identify the subtypes of FCon accurately, the clustering analysis (computed on MATLAB) was applied to process the anxiety/depressive rating (SAS, SDS, SAI, TAI) of 83 patients with FCon. Firstly, the scores of these four scales (SDS, SAS, SAI, and TAI) were normalized. Then, they were input to conduct agglomerative hierarchical clustering with a Euclidean distance metric and Ward’s minimum variance algorithm (Babbin et al., 2015; Grisanzio et al., 2018). The optimal number of clusters was determined using four standard methods including the silhouette coefficient (Rousseeuw, 1987), Calinski–Harabasz index (Wang and Xu, 2019), Davies–Bouldin index (Davies and Bouldin, 1979), and dendrogram (Van Soest et al., 2012); identifying the two-cluster was the optimal number of clusters (shown in Supplementary Figure 1). According to each cluster’s anxiety/depressive rating, one cluster was labeled FCAD and another cluster was labeled FCNAD. In addition, the k-medoids algorithm (Leiva-Valdebenito and Torres-Avilés, 2010) and fuzzy C-means algorithm (Bezdek, 1981) were applied to retest whether the partition of FCon subtypes is reliable (shown in Supplementary Information).

### MRI Acquisition

All MRI scans were performed in the morning between 9 and 10 AM to ensure consistency across assessment and minimize circadian influence. The experiments were carried out using a 1.5T GE (Signa HDXT, Milwaukee, WI, United States) scanner. First, the high-resolution structural image for each subject was acquired using T1-weighted three-dimensional magnetization-prepared rapidly obtained gradient-echo (MPRAGE) sequences (voxel size of 1 mm^3^ × 1 mm^3^ × 1 mm^3^) with the following parameters: repetition time (TR) = 9.14 ms, echo time (TE) = 2.93 ms, matrix size = 256 × 256, field of view (FOV) = 512 mm^2^ × 512 mm^2^, slice thickness = 1 mm, and 248 slices. Then, the gradient echo T2^∗^-weighted echo planar imaging sequence was used for acquiring RS-fMRI with the following parameters: TR = 2,000 ms, TE = 40 ms, matrix size = 64 × 64, flip angle = 90°, FOV = 256 mm^2^ × 256 mm^2^, slices = 29, and resolution = 4 mm^3^ × 4 mm^3^ × 4 mm^3^. The scan for RS-fMRI lasted 400 s, containing 200 echo-planar volumes. During the entire scanning procedure, subjects were asked to open their eyes and remain awake.

### Image Processing

Imaging data preprocessing was processed by using the Statistical Parametric Mapping (SPM) 12 toolkit^2^. The first five time points were removed to allow for magnetization equilibrium, and then slice-timing correction, frame-wise head movement correction, spatial normalization (echo-planar images were co-registered to everyone’s T1 anatomical image, spatially normalized to the template of the Montreal Neurological Institute and resampled to a voxel size of 3 mm^3^), and spatial smoothing (isotropic Gaussian kernel, full-width-at-half-maximum = 6 mm^3^) were performed (Zhang et al., 2015, 2018; Zhu et al., 2016; Li et al., 2018). There were no significant group differences on estimates of the subjects’ motion (*P* > 0.05) for mean/maximum frame-wise displacement calculated from six translation/rotation parameters obtained from the realignment process. Demeaning/detrending was carried out, and nuisance covariates including 24 head movement parameters, cerebrospinal fluid signals, and white matter signals were regressed out from blood oxygen level-dependent (BOLD) signals (Power et al., 2014; Chen et al., 2018b). fMRI time points that were severely affected by motion were removed using a “scrubbing method” (frame-wise displacement value > 0.5 mm and ΔBOLD of delta variation signal > 0.5%) (Power et al., 2014; Li et al., 2018, 2019; Zhang et al., 2018; Jin et al., 2019), and <5% of the time points were scrubbed from each subject. Band-pass filtering (0.01–0.08 Hz) was employed to reduce the effects of low-frequency drift/high-frequency noise by using the RS-fMRI Data Analysis Toolkit^3^.

### Network Construction

In graph theory, a network is constructed with two basic elements: nodes and edges. The nodes of the functional brain network represent brain regions, and network edges represent interregional resting-state FC (RSFC). Specifically, the human Brainnetome Atlas (Fan et al., 2016) was used to define network nodes by dividing the whole brain into 246 regions of interest (ROIs). Regional mean time series were obtained by averaging the fMRI time series over all gray matter voxels in each region, while Pearson’s correlation coefficients were calculated between the mean time series of each pair of regions. Fisher’s transformation was employed to convert correlation coefficients to *Z*-values for improving normality, thus a 246 × 246 *Z*-value symmetrical matrix was obtained for each subject. In order to ensure that the obtained networks had the same number of edges or wiring cost and minimized the number of spurious edges, each symmetrical matrix was thresholded into a binarized matrix with a fixed sparsity value that was defined as the total number of edges divided by the maximum possible number of edges in a network (Achard and Bullmore, 2007). Here, binarized matrices were obtained by repeatedly thresholding them over a range of sparsity levels (10%–30%) using an interval of 1% (Zhang et al., 2011; Meng et al., 2018).

### Network Analysis

To characterize the topological organization of the functional brain network, graph measures were calculated using the Graph Theoretical Network Analysis toolbox (GRETNA)^4^. Global parameters including the clustering coefficient (*C*_*p*_), shortest path length (*L*_*p*_), normalized clustering coefficient (γ), normalized shortest path length (λ), small-world-ness (σ), global efficiency (*E*_*glob*_), and local efficiency (*E*_*loc*_) were calculated. For regional parameters, nodal degree and nodal efficiency were applied. In this study, the modified greedy optimization algorithm (embedded in GRETNA) was used to estimate modular architectures by processing the undirected binary group-averaged matrices (thresholds ranged from 50 to 70% in 1% increments) (van den Heuvel and Sporns, 2011; de Reus and van den Heuvel, 2013) of the HC group. We constructed the group-averaged matrices in accordance with a method proposed by van den Heuvel and Sporns (2011). We computed the group average connectivity matrices by selecting all connections that were present in at least 50–70% of the set of 43 binarized brain networks from the HC group. These group average connectivity matrices were all based on the human Brainnetome Atlas. Then, parameters related to modular organization, which include modularity (*Q*), intra-module connectivity, inter-module connectivity, normalized within-module nodal degree, normalized within-module nodal efficiency, and participation coefficient, were calculated on modular architectures of the corresponding thresholds. The details and uses/interpretations of these network measures are described in the Supplementary Information. In order to eliminate the impact of sparsity selection, the area under the curve (AUC), which can provide a summarized scalar for topological characterization of the brain network, was introduced. We calculated the AUC value of all related network metrics over the corresponding sparsity range to explore the group effects and between-group differences in the functional brain networks among the FCAD, FCNAD, and HC groups. The integrated AUC metric has been used in previous brain network studies with a sensitivity of detecting topological alterations in brain disorders (Chen et al., 2018a).

### Statistical Analysis

SPSS (version 22; SPSS, Chicago) software was used for the demographic analysis. One-way ANOVA was applied to model the group effects (FCAD, FCNAD, and HC) on demographics, and a two-sample *t*-test was used for *post hoc* tests. Specifically, χ^2^ test was used to compare gender differences.

One-way ANOVA was employed to infer the group effects in functional network properties. In global network measures analyses, Bonferroni correction was applied as a multiple comparison correction; these network measures were considered significant for *P* < 0.05/7≈0.071 (seven graph theory metrics). For nodal and modular metrics analyses, FDR correction was used for multiple comparison correction because of too many nodes. After that, *post hoc* tests were applied to assess the between-group differences in these network metrics. Multiple linear regressions were employed to remove the effects of age/gender for each network metric before the one-way ANOVA and *post hoc* tests.

To further identify changes in pairwise RSFC in patient groups, we applied the Network-Based Statistic (NBS^5^), which is a validated, non-parametric statistical approach for controlling family wise error in connectome analyses (Zalesky et al., 2010). In brief, a primary cluster-defining threshold (*F* = 12, *P* < 0.05) was used in the F-statistic (one-way ANOVA) for comparing all three groups. This method was computed for each connection to define a set of supra threshold links among any connected components (number of edges). Then, the null distribution of the connected component size was empirically derived using a non-parametric permutation approach (10,000 permutations) to estimate the significance for each component. A corrected *P*-value was determined for each component using the null distribution of a maximal connected component size. After corrected weighted networks of all groups were entered into one-way ANOVA with the NBS approach (*P* < 0.05, NBS corrected for multiple comparisons), *post hoc* tests (employed a two-sample *t*-test) were performed to assess between-group differences in RSFC strength in the significant network obtained *via* ANOVA.

Finally, we conducted partial correlation analyses with age and gender as covariates to assess the association between network metrics/RSFC and clinical measures (difficulty of defecation, sensation of incomplete evacuation, abdominal distension/pain, SDS, SAS, SAI, and TAI) in FCAD/FCNAD groups. Bonferroni correction for multiple comparisons was executed: correlations for regional network measures were considered significant for *P* < 0.05/40 = 0.00125, correlations for inter-module connectivity were considered significant for *P* < 0.05/8 = 0.00625, and correlations for RSFC were considered significant for *P* < 0.05/64≈0.00078.

### Test–Retest Analysis

Reproducible studies have suggested that templates from different segmentations may result in diverse topological frameworks for graph analysis. We used the Automated Anatomically Labeled atlas (90 ROIs) and the “Willard” atlas (499 ROIs) (Fornito et al., 2010; Sanabria-Diaz et al., 2010; Mu et al., 2018)^6^ to construct networks in corresponding topological frameworks. The network metrics of the above brain functional networks were all calculated at multiple threshold values (from 10 to 30% using intervals of 1%), and AUC values were computed for these network metrics; moreover, the processes of calculation in retest networks were the same as those in human Brainnetome Atlas segmented networks. The network metrics included clustering coefficient (*C*_*p*_), shortest path length (*L*_*p*_), normalized clustering coefficient (γ), normalized shortest path length (λ), small-world-ness (σ), global efficiency (*E*_*glob*_), and local efficiency (*E*_*loc*_). The statistical analysis methods used in the retest were the same as those applied in the human Brainnetome Atlas segmented networks analysis.

## Results

### Demographic Characteristics

**FIGURE 1:**
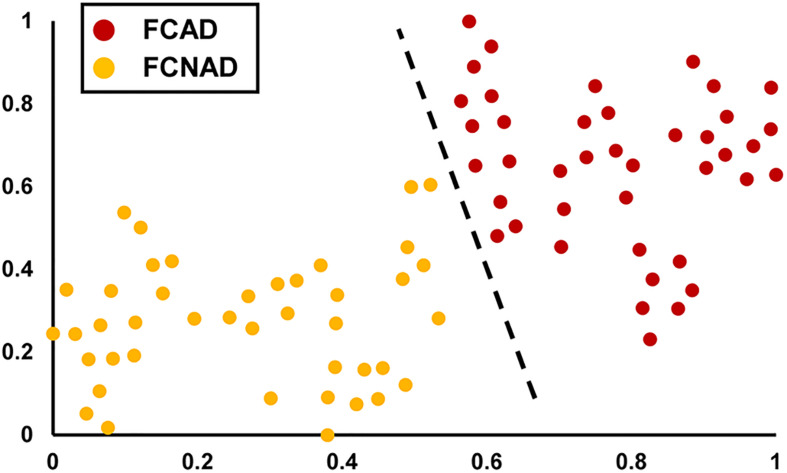
The unsupervised machine learning clustering algorithm identified a two-cluster solution (shown by t-SNE). The red points represent FCAD patients, and the orange points represent FCNAD patients. t-SNE, t-distributed stochastic neighbor embedding; FCAD, functional constipation associated with anxiety/depressive status; FCNAD, functional constipation without anxiety/depressive status.

In the current study, the clustering algorithm identified a two-cluster solution based on the measure of mental status (SDS, SAS, and STAI) of patients (Figure 1). According to each subtype’s anxiety/depressive rating, patients with FCon could be labeled as FCAD and FCNAD, respectively. Forty-one patients were classified as FCAD (age 38.61 ± 1.80 years, 10 males), and 42 patients were classified as FCNAD (age 42.76 ± 2.08 years, 16 males) (Figure 1 and Table 1).

There were no differences in age and gender among these three groups (*P* > 0.05; Table 1). There were significant group effects on depression (*F* = 167.154, *P* < 0.001), anxiety (*F* = 158.627, *P* < 0.001), state anxiety (SAI, *F* = 115.076, *P* < 0.001), and trait anxiety (TAI, *F* = 102.293, *P* < 0.001). *Post hoc* between-group comparisons showed that FCAD had higher SDS, SAS, and STAI ratings than HC and FCNAD (*P* < 0.001); although FCNAD had higher SDS, SAS, and STAI ratings than HC (*P* < 0.01), they did not reach a state of depression/anxiety in general (Table 1). FCAD and FCNAD were similar in difficulty of defecation, sensation of incomplete evacuation, and abdominal pain (*P* > 0.05) but scored differently in abdominal distension (*P* < 0.001; Table 1).

### Global Topological Organization of Functional Brain Networks

Whole brain network of the three groups showed typical features of small-world network architecture (σ > 1) in the defined sparsity range (from 10 to 30%, step = 1%) (Supplementary Figure 4).

**FIGURE 2:**
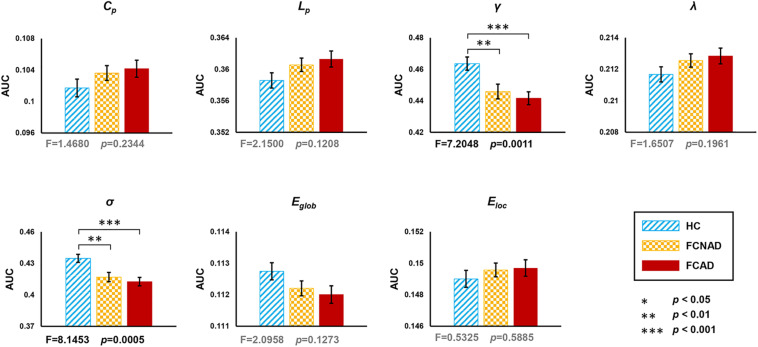
Group effects for global network metrics (AUC, after Bonferroni correction, *P* < 0.05/7≈0.0071) by using the human Brainnetome Atlas (HBA). Patients with FCAD/FCNAD showed significantly lower γ and σ than those in the HC group. AUC, area under the curve; *C*_*p*_, clustering coefficient; *L*_*p*_, shortest path length; γ, normalized clustering coefficient; λ, normalized shortest path length; σ, small-world-ness; *E*_*glob*_, global efficiency; *E*_*loc*_, local efficiency; FCAD, functional constipation associated with anxiety/depressive status; FCNAD, functional constipation without anxiety/depressive status; HC, healthy control.

In the current study, one-way ANOVAs showed significant group effects on γ (*F* = 7.205, *P* < 0.05, after Bonferroni correction) and σ (*F* = 8.145, *P* < 0.05, after Bonferroni correction; Figure 2). *Post hoc* tests showed that both FCAD and FCNAD had lower γ and σ than HC (*P* < 0.01). Moreover, there were no significant group effects on *C*_*p*_, *L*_*p*_, *E*_*glob*_, *E_*loc*_*, and λ (Figure 2).

### Regional Topological Organization of Functional Brain Networks

**FIGURE 3:**
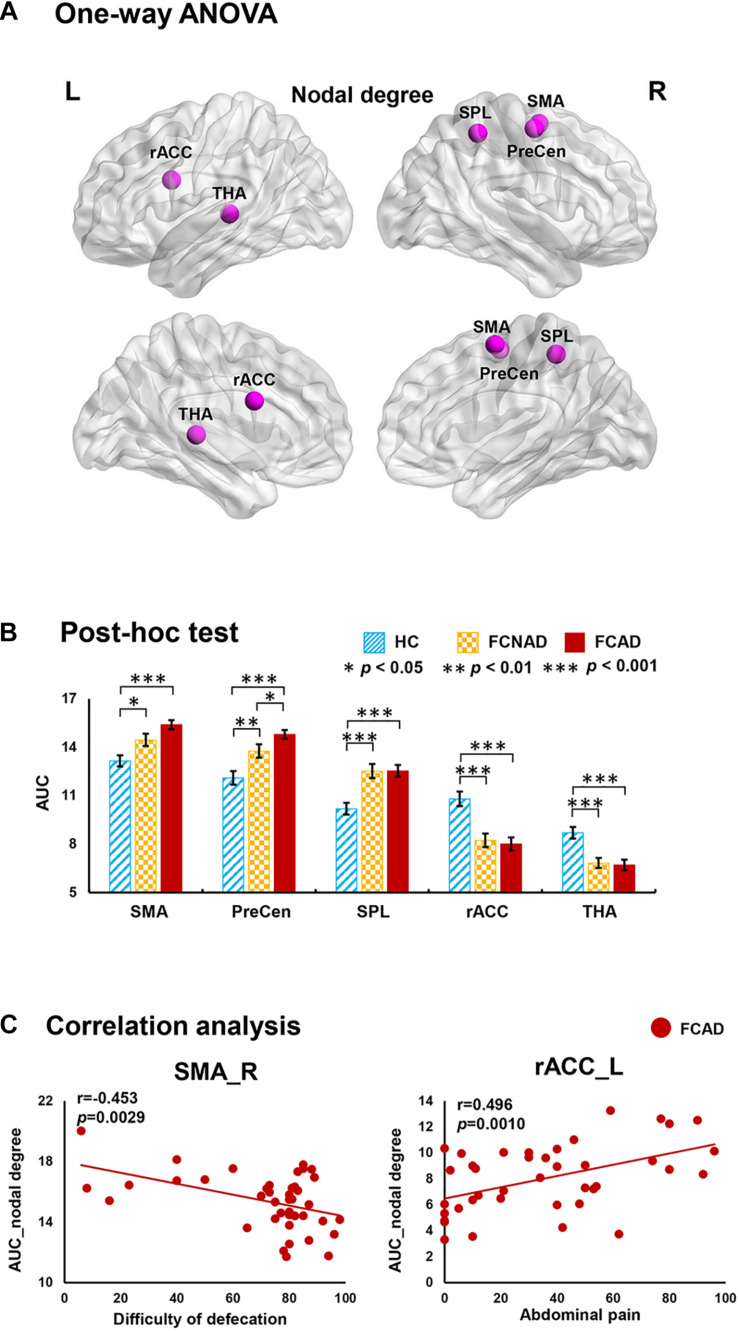
Group effects for nodal degree (AUC, *P*_*FDR*_ < 0.05) by using the human Brainnetome Atlas (HBA). There were significant differences in the rACC, THA, SMA, SPL, and PreCen **(A)**. Both FCAD and FCNAD had lower nodal degree than HC in the rACC and THA and higher nodal degree than HC in the SMA, PreCen, and SPL **(B)**. In the FCAD group, difficulty of defecation was negatively correlated with nodal degree in the SMA, and abdominal pain was significantly positively correlated with nodal degree in the rACC **(C)**. Images correspond to a 3D depiction of a see-through model of the areas in the brain, with darker colored ones indicating surface location and lighter colored ones indicating locations deeper in the brain. Nodes in pink represent significant group effect in nodal properties. AUC, area under the curve; SMA, supplementary motor area; PreCen, precentral gyrus; SPL, superior parietal lobule; rACC, rostral anterior cingulate cortex; THA, thalamus; FCAD, functional constipation associated with anxiety/depressive status; FCNAD, functional constipation without anxiety/depressive status; HC, healthy control; L, left; R, right.

**FIGURE 4:**
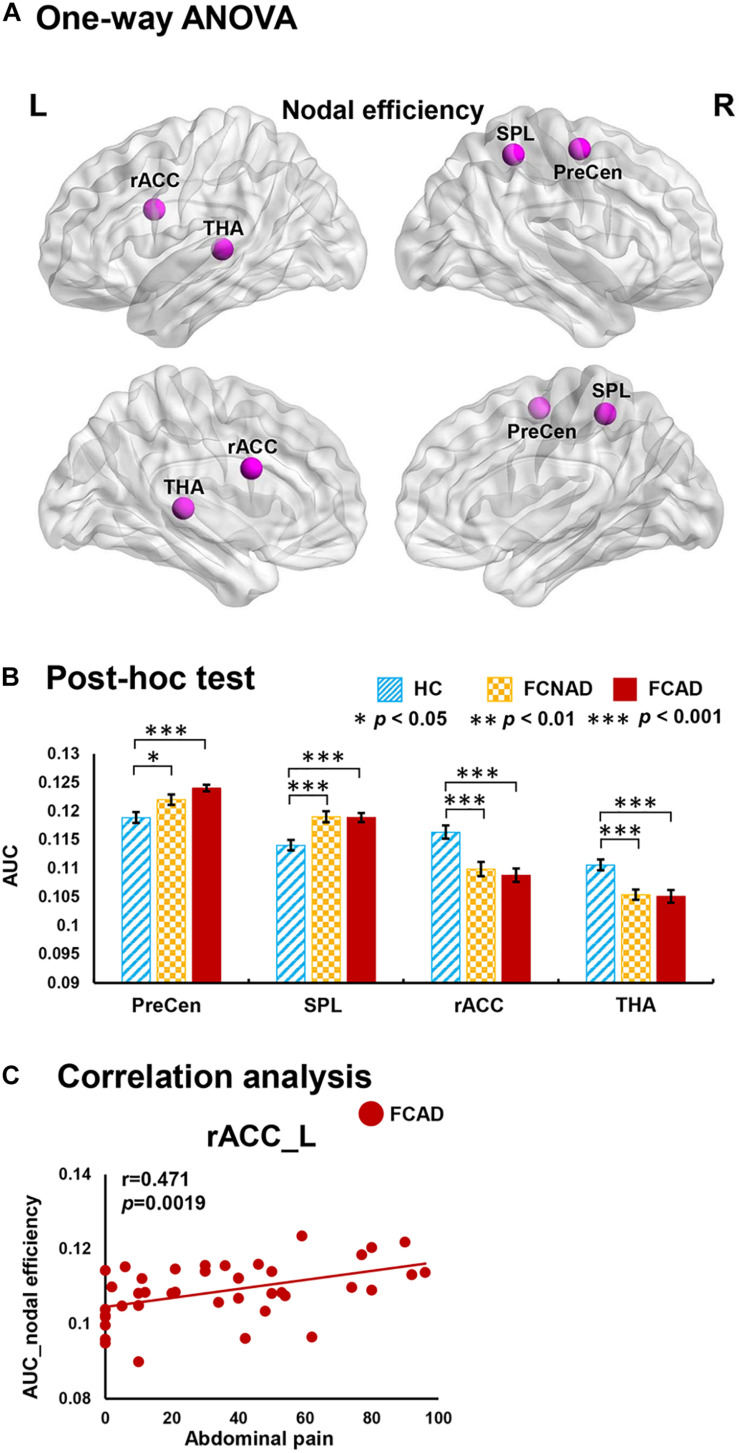
Group effects for nodal efficiency (AUC, *P*_*FDR*_ < 0.05) by using the human Brainnetome Atlas (HBA). There were significant differences in the rACC, THA, SPL, and PreCen **(A)**. Both FCAD and FCNAD had lower nodal efficiency than HC in the rACC and THA and higher nodal efficiency than HC in the PreCen and SPL **(B)**. In the FCAD group, abdominal pain was positively correlated with nodal efficiency in the rACC **(C)**. Images correspond to a 3D depiction of a see-through model of the areas in the brain, with darker colored ones indicating surface location and lighter colored ones indicating locations deeper in the brain. Nodes in pink represent significant group effect in nodal properties. AUC, area under the curve; PreCen, precentral gyrus; SPL, superior parietal lobule; rACC, rostral anterior cingulate cortex; THA, thalamus; FCAD, functional constipation associated with anxiety/depressive status; FCNAD, functional constipation without anxiety/depressive status; HC, healthy control; L, left; R, right.

There were significant group effects on nodal degree/efficiency (AUC) in the rACC, thalamus, SMA, PreCen, and superior parietal lobe (SPL) (*P*_*FDR*_ < 0.05; Figures 3A, 4A). *Post hoc* tests (Figures 3B, 4B) showed that both FCAD and FCNAD had lower nodal degree/efficiency than HC in rACC and thalamus (*P* < 0.001); both FCAD and FCNAD had higher nodal degree/efficiency than HC in the PreCen (*P* < 0.01), SMA (*P* < 0.05), and SPL (*P* < 0.001). Moreover, the nodal degree of PreCen in FCAD was higher than that in FCNAD (*P* < 0.05).

In the FCAD group, the nodal degree/efficiency (AUC) in rACC was positively correlated with abdominal pain (*r* = 0.496, *P* = 0.0010, Bonferroni-corrected, Figure 3C; *r* = 0.471, *P* = 0.0019, Figure 4C); difficulty of defecation had negative correlation with the nodal degree (AUC) of the SMA (*r* = −0.453, *P* = 0.0029; Figure 3C).

### Modular Topological Organization of Functional Brain Networks

**FIGURE 5:**
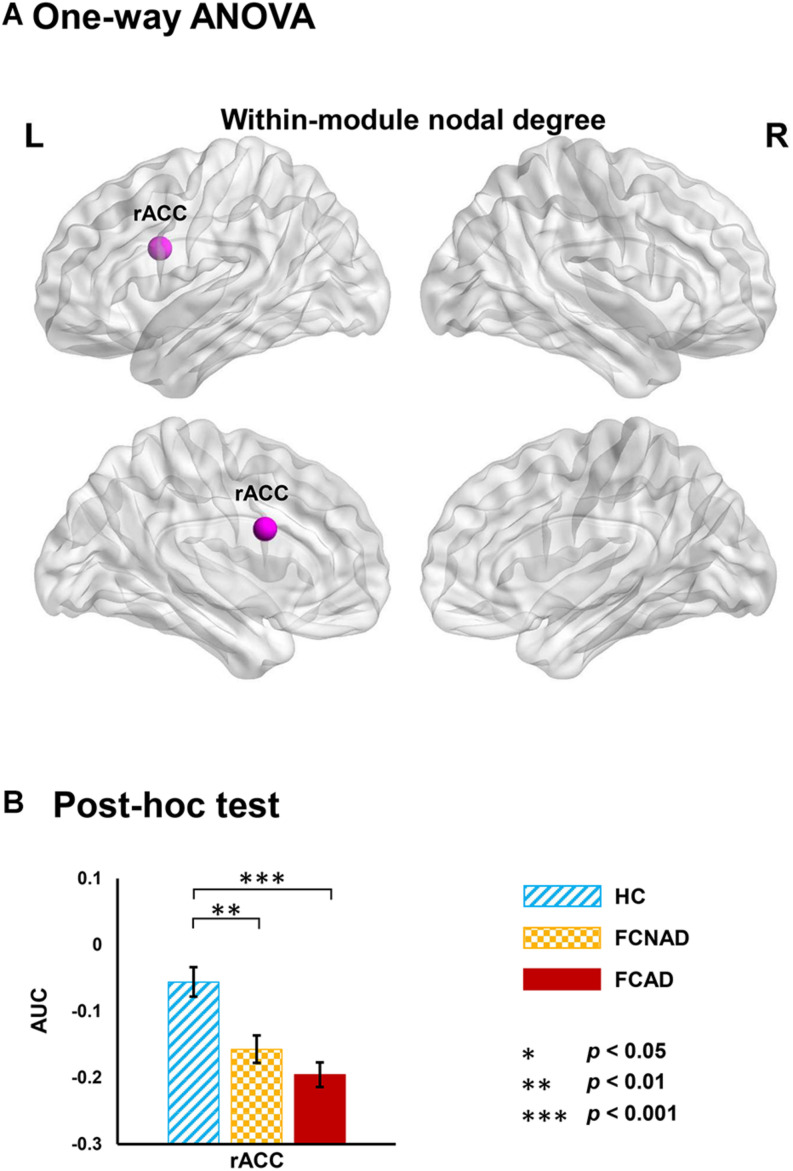
Group effects on within-module nodal degree (AUC, *P*_*FDR*_ < 0.05) based on human Brainnetome Atlas (HBA). **(A)** Significant difference in the rACC. **(B)** Both FCAD and FCNAD had lower within-module nodal degree than HC in the rACC. Images correspond to a 3D depiction of a see-through model of the areas in the brain, with darker colored ones indicating surface location and lighter colored ones indicating locations deeper in the brain. Nodes in pink represent significant group effect in nodal properties. AUC, area under the curve; rACC, rostral anterior cingulate cortex; FCAD, functional constipation associated with anxiety/depressive status; FCNAD, functional constipation without anxiety/depressive status; HC, healthy control; L, left; R, right.

Functional modules were identified based on the constructed binary group-averaged networks (sparsity ranged from 50 to 70% in 1% increments) of HC. Under the framework of these modular architectures, network metrics based on modular level (within-module nodal degree/efficiency and participant coefficient) were applied to further detect the identity of a node in the modular network structure in patients. Results only showed significant group effects on within-module nodal degree in rACC (*P*_*FDR*_ < 0.05; Figure 5A). *Post hoc* tests showed that both FCAD and FCNAD had lower within-module nodal degree than HC in rACC (*P* < 0.01; Figure 5B).

**FIGURE 6:**
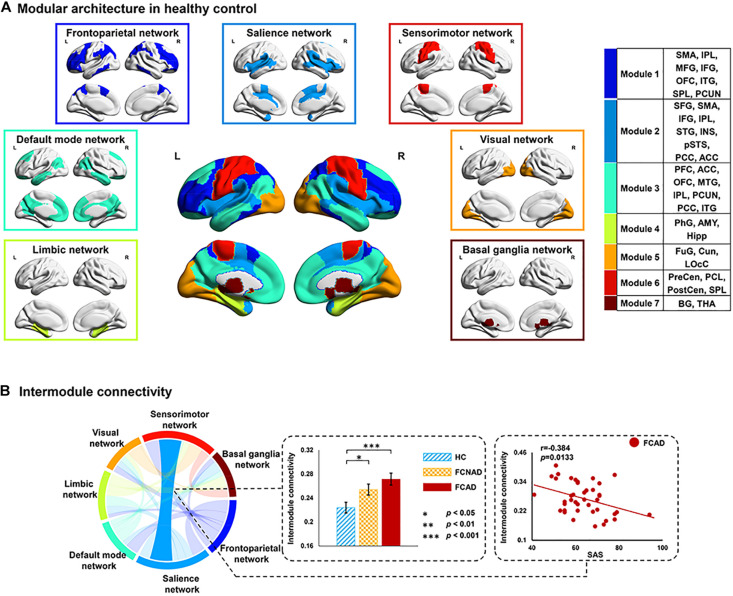
Spatial distribution of network modules and connectivity between modules. **(A)** Modular membership of healthy controls; seven modules were identified for the group-averaged functional network. **(B)** Group effect on inter-module connectivity between salience and sensorimotor networks (AUC, *P*_*FDR*_ < 0.05). Both FCAD and FCNAD had higher inter-module connectivity between salience and sensorimotor networks, which was negatively correlated with SAS in the FCAD group. AUC, area under the curve; SAS, ZUNG self-rating anxiety scale; FCAD, functional constipation associated with anxiety/depressive status; FCNAD, functional constipation without anxiety/depressive status; HC, healthy control; L, left; R, right.

Seven functional modules were detected in the HC group (*Q* = 0.6087) at a fixed network sparsity, including module 1 (frontoparietal network, 50 ROIs), module 2 (SN, 50 ROIs), module 3 (default mode network, 48 ROIs), module 4 (limbic network, 21 ROIs), module 5 (visual network, 22 ROIs), module 6 (SMN, 27 ROIs), and module 7 (basal ganglia network, 28 ROIs) (Supplementary Table 1). The functional modular architecture of the HC group is shown in Figure 6A. Intra-module connectivity and inter-module connectivity were analyzed by one-way ANOVA among FCAD, FCNAD, and HC under this modular architecture. Results showed that there was a significant group effect on the inter-module connectivity between the SN and SMN (*P*_*FDR*_ < 0.05; Figure 6B). *Post hoc* tests showed that the inter-module connectivity in both FCAD and FCNAD was higher than that in HC (*P* < 0.05). Moreover, SAS was negatively correlated with this inter-module connectivity in FCAD group (*r* = −0.384, *P* = 0.0133; Figure 6B).

### Altered Functional Network Connectivity

**FIGURE 7:**
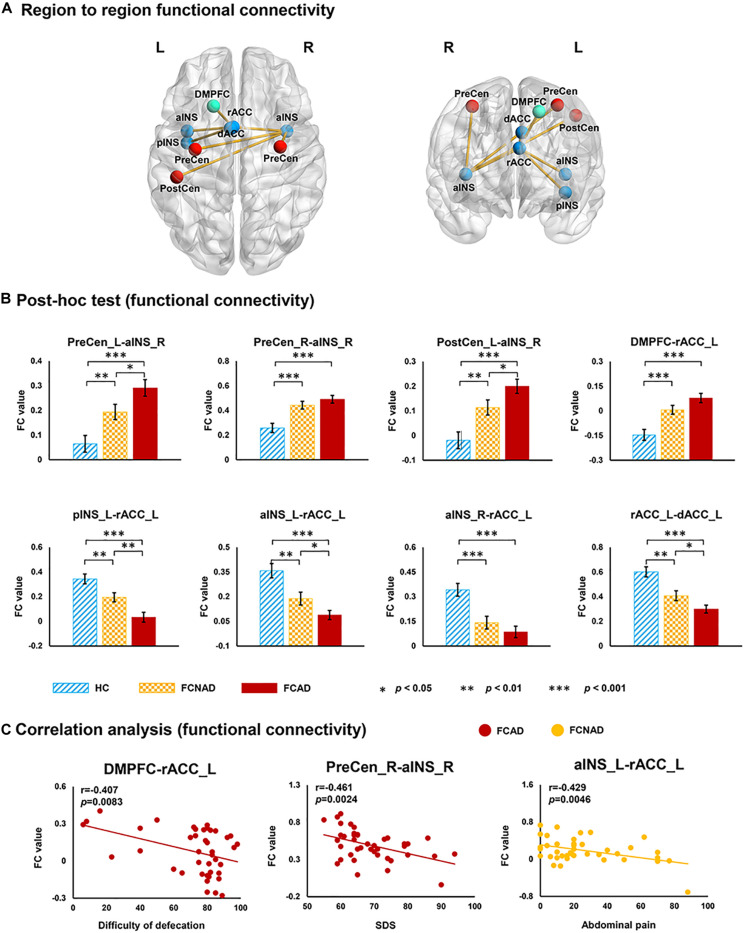
The subnetwork identified by network-based statistics (*P*_*NBS*_ < 0.05). **(A)** Group effects for region-to-region functional connectivity. **(B)** Network FCs within the SN in FCAD/FCNAD were lower than those in HC, whereas the opposite trend can be observed outside the SN. **(C)** In the FCAD group, difficulty of defecation was negatively correlated with RSFC of the DMPFC–rACC, and SDS was negatively correlated with RSFC of the PreCen-aINS. In the FCNAD group, abdominal pain had a negative correlation with RSFC of the aINS-rACC. rACC, rostral anterior cingulate cortex; dACC, dorsal anterior cingulate cortex; PreCen, precentral gyrus; aINS, anterior insula; pINS, posterior insula; PostCen, postcentral gyrus; DMPFC, dorsomedial prefrontal cortex; SDS, ZUNG self-rating depressive scale; FCAD, functional constipation associated with anxiety/depressive status; FCNAD, functional constipation without anxiety/depressive status; HC, healthy control; L, left; R, right.

A subnetwork identified by using the NBS method, which consisted of nine nodes and eight connections, showed a significant group effect among FCAD, FCNAD, and HC groups (*F* = 12, *P*_*NBS*_ < 0.05; Figure 7A). This subnetwork was centered on the left rACC, and five connections were associated only with the left rACC. More specifically, there was one connection located between the dorsomedial prefrontal cortex [DMPFC, default mode network (DMN)] and four connections with the brain regions in the SN [bilateral anterior insula (aINS), left dorsal ACC (dACC), and left posterior INS (pINS)]. Moreover, there were three other brain regions [bilateral PreCen, left postcentral gyrus (PostCen)], which belong to the SMN, and they were connected with the right aINS in the subnetwork. Subsequent *post hoc* tests (Figure 7B) found that the RSFCs of the left/right PreCen–right aINS, left PostCen–right aINS, and DMPFC–left rACC in FCAD/FCNAD were higher than those in HC (*P* < 0.01); the other altered RSFCs in FCAD/FCNAD were lower than those in HC (*P* < 0.01). In the FCAD group, the RSFC of the DMPFC–left rACC was negatively correlated with difficulty of defecation (*r* = −0.407, *P* = 0.0083); SDS had a negative correlation with the RSFC of the right PreCen–right aINS (*r* = −0.461, *P* = 0.0024). Meanwhile, in the FCNAD group, there was a negative correlation between abdominal pain and RSFC of the left aINS–left rACC (*r* = −0.429, *P* = 0.0046) (Figure 7C).

### Reproducibility of Findings

Using the “Willard” atlas (499 ROIs), one-way ANOVAs exhibited significant group effects on γ (*F* = 5.265, *P* < 0.05, Bonferroni-corrected) and σ (*F* = 6.122, *P* < 0.05, Bonferroni-corrected), and *post hoc* tests showed that FCAD/FCNAD had lower γ and σ than those in HC (*P* < 0.01; Supplementary Figure 5). Applying the automated anatomically labeled template (90 ROIs), one-way ANOVAs showed a significant group effect on σ (*F* = 6.913, *P* < 0.05, Bonferroni-corrected), while *post hoc* tests exhibited σ in FCAD/FCNAD, which was lower than that in the HC group (*P* < 0.05; Supplementary Figure 6). These findings were generally consistent with results calculated by using the human Brainnetome Atlas, suggesting the reproducibility of the current study.

## Discussion

In the current study, we investigated the differences in the intrinsic brain functional networks at multiple levels of the network architecture between FCAD and FCNAD by combining RS-fMRI with graph theory approach. Demographic information showed that FCAD had a more serious constipation symptom (i.e., abdominal distension) than FCNAD in the clinics. One-way ANOVAs showed significant group effects on normalized clustering coefficient (γ) and small-world-ness (σ). Differences in regional level mainly were located in the rACC, PreCen, thalamus, SMA, and SPL. Alteration in within-module nodal degree highlighted the rACC-related lower information integration within the SN in FCAD/FCNAD. Results also revealed a significant group effect on inter-module connectivity between the SN and SMN and negative correlation between this inter-module connectivity and SAS. In the FCNAD group, the RSFC (left aINS–left rACC) was correlated only with constipation symptoms. While in the FCAD group, the RSFC (DMPFC–left rACC and right PreCen–right aINS) were correlated not only with constipation symptoms but also with depressive ratings (SDS), suggesting different gut–brain interactions between FCAD and FCNAD. In addition, the altered RSFC in FCAD/FCNAD identified by NBS further highlighted the core position of rACC in the brain network. Findings indicated that there were brain functional differences mainly within and between the SN and SMN in FCAD/FCNAD.

### Lower Small-World Properties of the Brain Network in Both FCAD and FCNAD

Results revealed that the brain functional networks in FCAD, FCNAD, and HC groups all had typical small-world properties. Both FCAD and FCNAD groups showed lower normalized clustering coefficient and small-world-ness compared with HC. As the clustering coefficient measures the ability for specialized processing to occur within densely interconnected groups of brain regions (Vecchio et al., 2014), the lower value suggested decreased local FC of the brain network in FCAD/FCNAD. This would lose the optimal balance of local network information processing and efficient overall routing with shortcuts of brain networks, leading to lower small–world-ness in FCAD/FCNAD. Previous study on functional dyspepsia (a typical subtype of functional gastrointestinal disorder) reported decreased functional connections in the ACC and thalamus, indicating altered local FC related to gastrointestinal symptoms (Nan et al., 2014). The results of the current study were consistent with the previous report; observations of the lower within-module nodal degree of rACC in both FCAD and FCNAD also supported that the lost optimal balance of high synchronization ability and fast information transmission of the brain network were associated with FCon.

### Rostral Anterior Cingulate Cortex-Related Alterations in the Salience Network

The SN, as an important large-scale brain network involved in integrating sensory input to attend salient stimuli, guides attention and recruits appropriate functional brain–behavior networks to modulate behavior (Menon and Uddin, 2010; Peters et al., 2016). For the SN, the current study showed that there was a significantly lower within-module nodal degree in rACC, reflecting that rACC had decreased ability of information integration inside the SN in FCAD/FCNAD. The ACC belongs to the inhibitory control circuit of the brain (Szekely et al., 2017). It critically participated in emotional processing modulation, including negative emotional processing and modulation of pain (Naliboff et al., 2001; Petrovic and Ingvar, 2002). In the current study, NBS-based findings showed lower RSFC between rACC and other brain regions (i.e., dACC, aINS, and pINS) within the SN in the FCAD/FCNAD groups, which was supported by these findings. Moreover, rACC-related alterations in the SN were also widely reported in other functional gastrointestinal disorder studies (Hong et al., 2013; Mayer et al., 2015). The dACC is involved in multiple functions, such as emotional, cognitive, and motor functions (Heilbronner and Hayden, 2016). It is a central station for processing top–down and bottom–up stimuli and assigning appropriate control to other brain areas (Dockstader et al., 2014). Much evidence supported that there was a tight functional association between rACC and aINS, which was crucial for the regulation of psychological activities (i.e., emotion awareness) (Medford and Critchley, 2010). The aINS is involved in the awareness of the unpleasant feeling associated with pain, and the ACC is involved in the marshaling of a response to this unpleasantness (Medford and Critchley, 2010). The pINS activates the primary cortical area of termination of an interoceptive afferent pathway during nociceptive stimuli and function as a comparator between expected and actual sensation (Uddin et al., 2017; Benarroch, 2019). Previous study with sensory testing showed that pain thresholds were elevated after lesions involving the posterior insula (Greenspan and Winfield, 1992). For the suprathreshold stimuli, individuals with INS lesions had been reported to exhibit a reduced appreciation of the meaning and significance of noxious stimuli (Berthier et al., 1988). Another study on FCon also reported the altered interaction between ACC and other regions (i.e., INS, OFC, and HIPP), which belong to the emotional–arousal network, reflecting altered emotional processes and integration of somatic and visceral information (Zhu et al., 2016). Therefore, these rACC-related lower RSFCs within the SN reflected the altered emotional and homeostatic responses to salient stimuli input in patients with FCAD/FCNAD (Seeley, 2019). Interestingly, the RSFCs (i.e., left pINS–left rACC, left aINS–left rACC, left rACC–left dACC) within the SN in the FCAD group were weaker than those in the FCNAD group.

In the FCAD group, the positive correlation between the nodal degree/efficiency (AUC) of rACC and abdominal pain indicated that the more severe the abdominal pain, the higher the nodal degree/efficiency in rACC in FCAD. Previous studies in patients with irritable bowel syndrome also found that altered brain activity of rACC was associated with abdominal pain (Hobson and Aziz, 2004; Drossman, 2005), and these studies had linked fear or other negative emotions (e.g., anxiety) to visceral pain (Ringel et al., 2003; Drossman, 2005). However, the association between within-module nodal degree of rACC and abdominal pain in FCAD was not observed in the current study. These findings indicated that the rACC as a part of the inhibitory control circuit needs to have more functional connections with other brain regions outside the SN for coping with pain. Results also showed altered RSFC between rACC and DMPFC (one of the core regions of DMN). DMPFC and rACC are part of the affective network, which is frequently activated during anticipation of aversive events (rather than during the actual experience) and during normal and pathological anxiety (Mayer et al., 2005). Thus, the negative correlation between this RSFC and difficulty of defecation suggested that FCAD needs higher functional association of these two regions to reduce unpleasantness associated with aversive events and anxiety.

### Alterations Related to Sensorimotor Network and Altered Topological Characteristics of Supplementary Motor Area and Thalamus

The SMN receives sensory input from the periphery. It is important for awareness of body sensations and generation of appropriate motor responses (Mayer et al., 2015). In the current study, the result of inter-module connectivity showed that there were altered FCs between the SN and SMN in FCAD/FCNAD. By employing NBS, a part of higher inter-module connectivity (SN–SMN) was identified between aINS and PreCen/PostCen. PreCen and PostCen are the core regions of the SMN. PreCen is a prominent structure of the primary motor cortex (Zhu et al., 2016), mainly contributing to the control of movement. As for PostCen, it is mainly responsible for processing visceral stimulation (Nan et al., 2020). The altered inter-network RSFCs related to these two functional networks were also reported in other studies on functional gastrointestinal disorders, which were associated with altered integrations and processing of somatic and visceral information (Zhu et al., 2016; Nan et al., 2020). While the negative correlation between SAS and inter-module connectivity of the SN-SMN in FCAD might reflect the self-protection/regulation mechanisms of the brain (Heatherton, 2011), that is, by weakening the interaction between these two networks to avoid more intense responses to stronger anxiety status in patients with FCAD. Moreover, the current study further found that SDS had a negative correlation with the RSFC of the right PreCen–right aINS in FCAD, indicating that the interaction between the aINS and PreCen would be weaker during the aggravated depressive status, thus leading to inappropriate motor responses, which would aggravate constipation symptoms. These findings suggested that the anxiety/depression status of patients with FCAD was associated with altered communication between the SN (especially in aINS) and SMN. Furthermore, the higher nodal degree/efficiency (AUC) in the PreCen reflected the important role of the PreCen in the brain network of patients with FCAD/FCNAD (more important in patients with FCAD), indicating that constipation altered the ability to control bowel movements (Jin et al., 2019).

The SMA is part of the primate cerebral cortex regulating motor function (Schell and Strick, 1984) and is sensitive to visceral sensory stimuli (Guleria et al., 2017). Results showed a higher nodal degree of the SMA in FCAD/FCNAD, which reflected that the SMA had more connections with other brain regions and was consistent with the altered FC density in patients with irritable bowel syndrome (Weng et al., 2017). Combined with the negative correlation between nodal degree of the SMA and difficulty of defecation in FCAD group and a similar difficulty of defecation between the FCAD and FCNAD groups in the clinics, these findings suggested that the SMA in FCAD had to interact with more brain regions than FCNAD to complete defecation. Moreover, the more brain regions involved, the smoother defecation would be.

Thalamus is a central region relaying/integrating/transmitting various information with multiple cortical areas (Rouiller et al., 1999; Herrero et al., 2002; Jones et al., 2006; Keszthelyi et al., 2012) and plays a critical role in perceptual processing (Sherman and Guillery, 2006). Studies on irritable bowel syndrome revealed that the thalamus was more critical for controlling sensory information in irritable bowel syndrome patients (Labus et al., 2014) and was also activated during rectal distension (Mayer et al., 2009; Larsson et al., 2012). The lower nodal degree/efficiency in the thalamus in FCAD/FCNAD suggested that there was altered integration of visceral sensory signals between the thalamus and other brain regions (Jones et al., 2006; Al Omran and Aziz, 2014). In addition, the nodal degree/efficiency of SPL in FCAD/FCNAD was higher than that in HC, reflecting altered brain responses to movement/sensory stimuli in patients (Cavanna and Trimble, 2006).

### Limitations

Due to the strict exclusion criteria, we did not obtain a larger cohort for FCAD, FCNAD, and HC groups, which limited the generalizability and statistical power of our observations. Moreover, we did not collect the educational information of the subjects.

## Conclusion

The current study aimed to investigate differences in FC of resting-state networks between FCAD and FCNAD. Altered network organization in FCAD and FCNAD was examined at three levels (global, regional, and modular) by employing RS-fMRI combined with graph theoretical analysis. Results showed that there was altered FC within and between the SN and SMN in FCAD/FCNAD. In the FCNAD group, the RSFCs were only correlated with constipation symptoms. While in the FCAD group, the topological characteristics and RSFCs were correlated not only with constipation symptoms but also with depressive ratings. These findings indicate the differences in FC of the SN–SMN between FCAD and FCNAD and provide neuroimaging evidence based on brain function that portrays important clues for improving new treatment strategies.

## Data Availability Statement

The data analyzed in this study is subject to the following licenses/restrictions: the concern over patient privacy. Requests to access these datasets should be directed to SD, 283507727@qq.com.

## Ethics Statement

The studies involving human participants were reviewed and approved by the experimental protocol was approved by the Institutional Review Board of Xijing Hospital and was registered in the Chinese Clinical Trial Registry Center as ChiCTR-OOB-15006347 (http://www.chictr.org.cn). The experiments were conducted in accordance with the Declaration of Helsinki. The patients/participants provided their written informed consent to participate in this study.

## Author Contributions

YZ, YN, and GC contributed to the study concept and design. SD and YN performed the diagnosis. SD, ZT, ZJ, LZ, YH, WZ, and JW contributed to the acquisition of fMRI data. LL contributed to the data analysis and interpretation of findings. LL and GL drafted the manuscript. GL, KD, and YZ provided critical revision of the manuscript for important intellectual content.

## Conflict of Interest

The authors declare that the research was conducted in the absence of any commercial or financial relationships that could be construed as a potential conflict of interest.

## Footnotes

1 http://www.chictr.org.cn

2 http://www.fil.ion.ucl.ac.uk/spm

3 http://resting-fmri.sourceforge.net

4 https://www.nitrc.org/projects/gretna

5 https://www.nitrc.org/projects/nbs/

6 http://findlab.stanford.edu/functional_ROIs.html
