# Supplementary material for: Altered Functional Connectivity Within and Between Salience and Sensorimotor Networks in Patients With Functional Constipation
Source: Front Neurosci. 2021 Mar 11;15:628880. doi: 10.3389/fnins.2021.628880 (PMC7991789; doi:10.3389/fnins.2021.628880)
Supplement: Supplementary file 1 [file Data_Sheet_1.DOCX]

**Supplementary Information**

**Materials and Methods**

*Stability of clustering solutions*

To ensure our clustering solution was not a specific result of our clustering method, we applied k-medoids(Wu, 2011) and fuzzy C-means (FCM) (Bezdek, 1981)as a validation method in the current study (the corresponding optimal number of clusters is shown in **Supplementary Figure 2-3**, respectively). The Adjusted Rand Index (introduced below) was employed to test the similarity between each clustering solution (k-medoids and FCM) compared to the original clustering solution (hierarchical clustering). In the results, individuals were largely assigned to the same cluster group for both k-medoids (Adjusted Rand Index: 0.952) and FCM (Adjusted Rand Index: 0.905) when compared to the hierarchical clustering solution. The similarity in performance indicates that the clustering solution found using the hierarchical centroids was stable.

*Adjusted Rand Index*

*The Adjusted Rand Index*is the corrected-for-chance version of the Rand index(Rand, 1971; Hubert and Arabie, 1985; Vinh et al., 2009).Such a correction for chance establishes a baseline by using the expected similarity of all pair-wise comparisons between clusterings specified by a random model. Traditionally, the Rand Index was corrected using the Permutation Model for clusterings (the number and size of clusters within a clustering are fixed, and all random clusterings are generated by shuffling the elements between the fixed clusters). However, the premises of the permutation model are frequently violated; in many clustering scenarios, either the number of clusters or the size distribution of those clusters vary drastically. For example, consider that in K-means the number of clusters is fixed by the practitioner, but the sizes of those clusters are inferred from the data. Variations of the adjusted Rand Index account for different models of random clusterings (Gates and Ahn, 2017).

In the current study, the Adjusted Rand index of retested clustering solutions (k-medoids and fuzzy C-means) were calculated based on the scikit-learn, a software machine learning library (Pedregosa et al., 2011).

*Network metrics*

In order to characterize the topological organization of the functional brain networks, we employed common graph metrics to analyze the brain binary networks at each threshold value. The graph metrics we used were as follows: clustering coefficient ($C_{p}$), shortest path length ($L_{p}$), normalized clustering coefficient (*γ*), normalized shortest path length (*λ*), small-worldness (*σ*), global efficiency ($E_{glob}$) and local efficiency ($E_{loc}$),nodal degree, nodal efficiency, modularity (*Q*), intra-module connectivity ($C_{s}$), inter-module connectivity ($C_{s,t}$), normalized within-module nodal degree (WD), normalized within-module nodal efficiency (WE) and participation coefficient (PC). Then, one-way ANOVA analyses were employed to infer group effects of functional network metrics. Post-hoc two sample *t-*tests were employed to infer the between-group differences in these network measures among FCAD, FCNAD and HC groups. The details and interpretations of these network measures are described below.

*The clustering coefficient* quantifies the extent of local inter-connectivity or cliquishness of a network. For a given node *i*, $C_{i}$is defined as:

$$C_{i}=\frac{2e_{i}}{k_{i}(k_{i}-1)}$$

where $k_{i}$is the degree of node *i*, and $e_{i}$ is the number of actual existing connections between the nearest neighbors of node *i*.$C_{i}$ is a ratio of the total number of edges divided by the maximum possible number of edges between the nearest neighbors of node *i*. The mean clustering coefficient of network $C_{p}$ is the average of the clustering coefficient over all nodes in a network(Watts and Strogatz, 1998):

$$C_{P}=\frac{1}{N}C_{i}$$

*The shortest path length* reflects the mean minimal travel path between any pair of nodes(e.g., node *i* and node *j*) in the network. The shortest path length of a network is defined as:

$$L_{p}=\frac{1}{N(N-1)}\sum_{i\neq j\in G} L_{ij}$$

where *N* is the number of nodes in the network, and $L_{ij}$is defined as the shortest path length between node *i* and node *j* in the network. The shortest path length of a network quantifies the average number of connections between nodes along the shortest paths(Zhu et al., 2018).

To compute the small-world properties of the network, the values of the clustering coefficient ($C_{p}$) and shortest path length ($L_{p}$) were compared with 100 matched random networks. The random networks preserve the same number of nodes, edges and degree distribution as real networks (Maslov and Sneppen, 2002). Furthermore, the normalized clustering coefficient (*γ*) and normalized shortest path length (*λ*) were calculated as follows:

$$\gamma=\frac{C_{p}^{real}}{C_{p}^{rand}}$$

$$\lambda=\frac{L_{p}^{real}}{L_{p}^{rand}}$$

where $C_{p}^{rand}$ and $L_{p}^{rand}$ are the mean clustering coefficient and shortest path length of 100 matched random networks. A network is considered to have small-world properties if *γ*>1 and *λ*~1. Then, these two parameters were unified into a simple quantitative parameter, small-worldness (*σ*). The small-worldness of a network is defined as:

$$\sigma=\frac{\gamma}{\lambda}$$

A real network is said to be small-world if *σ*> 1, and has a stronger small-world property when the value of *σ* is higher.

*The global efficiency* ($E_{glob}$) of network G measures the ability of parallel information transmission in the network (Latora and Marchiori, 2001),which is computed as follows:

$$E_{glob}(G)=\frac{1}{N(N-1)}\sum_{i\neq j\in G} \frac{1}{L_{ij}}$$

where $L_{ij}$ is the shortest path length between node *i* and node *j* in network G.

*The local efficiency* ($E_{loc}$) of network G measures how much the network is fault tolerant and shows the capability of information transfer in each subgraph when node *i* is removed (Latora and Marchiori, 2001), which is defined as:

$$E_{loc}(G)=\frac{1}{N}\sum_{i\in G} E_{glob}(G_{i})$$

where $E_{glob}$ is the global efficiency of the network, and $G_{i}$denotes the subgraph composed of the nearest neighbors of node *i*.

*The nodal degree* is defined as the number (binary graph) or the total connectivity strength (weight graph) of all connections that link to a node, reflecting the centrality of this node in the network.

*The nodal (regional) efficiency* ($E_{nodal}$) measures the mean shortest path length between a given node *i* and all of the other nodes in the network(A chard and Bullmore, 2007),which is defined as follows:

$$E_{nodal}=\frac{1}{N-1}\sum_{i\neq j\in G} \frac{1}{L_{ij}}$$

where $L_{ij}$ is the shortest path length between node *i* and node *j* in network G.

Modular organization is one of the important features of the brain network. A module is topologically defined as a subset of nodes tightly connected within the modules but sparsely connected to nodes in other modules(Meunier et al., 2010).

In the current study, the community (module) structures were examined by applying the modified greedy optimization algorithm (embedded in GRETNA) to process group-averaged brain networks of the HC group at different thresholds. The modified greedy optimization algorithm was a modified hierarchical agglomeration algorithm for detecting community structure(Clauset et al., 2004; Danon et al., 2006). The group-averaged matrix was in accordance with a method proposed by van den Heuvel and Sporns(van den Heuvel and Sporns, 2011). For unweighted matrices, from the set of N (number of subjects) individual binarized connectivity matrices M, a group average connectivity matrix M group was computed by selecting all connections that were present in at least T (a selected threshold, e.g. 50%) of the group of subjects. The following computations of modular parameters were based on these community structures.

*The modularity Q* of the network(Newman and Girvan, 2004)is defined as:

$$Q=\frac{1}{4m}\sum_{ij} (A_{ij}-\frac{k_{i}k_{j}}{2m})s_{i}s_{j}$$

where $k_{i}$ and $k_{j}$ are the degree of node *I* and node *j* respectively, $A_{ij}$ is the number of connections between node *i* and node *j*, and *m* is the total number of edges in the network. If the network could be divided into two groups, $s_{i}$=1 suggests that the *ith* node belongs to group 1 and $s_{i}$=－1 suggests that the *ith* node belongs to group 2. Modularity *Q* quantifies the difference between the number of intra-module edges of the actual network and that of the random network(Newman, 2006). The *Q* approaching 0 means that the nodes are partitioned randomly or all nodes are the subset of the same module, whereas a higher value of *Q* suggests a significant modular structure deviating from random networks(Newman, 2006).In practice, the value of *Q* for the network with a powerful modular structure typically ranges from 0.3 to 0.7(Newman and Girvan, 2004).

In order to assess the inter- and intra-modular connectivity at both modular and nodal (regional) levels at the corresponding module architecture, we employed several relevant parameters in the current study. At the module level, intra-module connectivity and inter-module connectivity were calculated as follows:

*Intra-module connectivity* ($C_{s}$) of module *s* is computed as follows:

$$C_{s}=\frac{\sum_{i,j\in s} \varepsilon_{ij}}{N_{s}(N_{s}-1)}$$

where$N_{s}$is the number of nodes within module *s*, and $\varepsilon_{ij}$represents the edges which actually exist within module*s*.

*Inter-module connectivity* ($C_{s,t}$) between module *s* and module *t* is defined as:

$$C_{s,t}=\frac{\sum_{i\in s,j\in t} \varepsilon_{ij}}{N_{s}\times N_{t}}$$

where $N_{s}$ is the number of nodes within module *s* and $N_{t}$ is the number of nodes within module *t*, and $\varepsilon_{ij}$includes the edges which actually exist between module *s*and module *t*.

Meanwhile, we also employed network metrics at the nodal (regional) level to analyze the modular organization of the network:

*Normalized within-module nodal degree (WD)* is defined as follows:

$$WD_{i}=\frac{k_{i}-\overline{k_{s}}}{\sigma_{k_{s}}}$$

where $k_{i}$ is the number of links of node *i* to other nodes within module *s* and $\overline{k_{s}}$ is the average of the nodal degree over all of the nodes within module *s*, and$\sigma_{k_{s}}$is the standard deviation of the nodal degree over all of the nodes within module *s*. The normalized within-module nodal degree measures how ‘well connected’ node *i* is to other nodes in the module(Guimera and Amaral, 2005).

*Normalized within-module nodal efficiency (WE)* is defined as follows:

$$WE_{i}=\frac{e_{i}-\overline{e_{s}}}{\sigma_{e_{s}}}$$

where $e_{i}$ is the nodal efficiency of node *i* within module *s* and $\overline{e_{s}}$ is the mean nodal efficiency over all nodes within module *s*, and $\sigma_{e_{s}}$is the standard deviation of nodal efficiency over all of the nodes within module *s*.

*Participation coefficient (PC)* is defined as follows:

$$PC_{i}=1-\sum_{s=1}^{N_{M}} (\frac{k_{is}}{k_{i}})^{2}$$

where $N_{M}$ is the number of modules and $k_{is}$ is the number of edges between node *i* and module *s*, and $k_{i}$ is the total degree of node *i*. The participation coefficient of a node is used as the index for inter-module connection densities(Guimera and Nunes, 2005).

**References**

Achard, S., and Bullmore, E. (2007). Efficiency and cost of economical brain functional networks. *PLoS Comput Biol* 3, e17. doi: 10.1371/journal.pcbi.0030017.

Bezdek, J.C. (1981). *Pattern recognition with fuzzy objective function algorithms*. New York: Plenum Press.

Clauset, A., Newman, M.E., and Moore, C. (2004). Finding community structure in very large networks. *Phys Rev E Stat Nonlin Soft Matter Phys* 70, 066111. doi: 10.1103/PhysRevE.70.066111.

Danon, L., Díaz-Guilera, A., and Arenas, A. (2006). The effect of size heterogeneity on community identification in complex networks. *Journal of Statistical Mechanics: Theory and Experiment* 2006, P11010-P11010. doi: 10.1088/1742-5468/2006/11/P11010.

Gates, A.J., and Ahn, Y. (2017). The Impact of Random Models on Clustering Similarity. doi.

Guimera, R., and Amaral, L.A. (2005). Cartography of complex networks: modules and universal roles. *J Stat Mech* 2005, nihpa35573. doi: 10.1088/1742-5468/2005/02/P02001.

Guimera, R., and Nunes, A.L. (2005). Functional cartography of complex metabolic networks. *Nature* 433, 895-900. doi: 10.1038/nature03288.

Hubert, L., and Arabie, P. (1985). Comparing partitions. *J. Classif.* 2, 193-218. doi: 10.1007/BF01908075.

Latora, V., and Marchiori, M. (2001). Efficient behavior of small-world networks. *Phys. Rev. Lett.* 87, 198701. doi: 10.1103/PhysRevLett.87.198701.

Maslov, S., and Sneppen, K. (2002). Specificity and stability in topology of protein networks. *Science* 296, 910-3. doi: 10.1126/science.1065103.

Meunier, D., Lambiotte, R., and Bullmore, E.T. (2010). Modular and hierarchically modular organization of brain networks. *Front Neurosci* 4, 200. doi: 10.3389/fnins.2010.00200.

Newman, M.E. (2006). Modularity and community structure in networks. *Proc Natl Acad Sci U S A* 103, 8577-82. doi: 10.1073/pnas.0601602103.

Newman, M.E., and Girvan, M. (2004). Finding and evaluating community structure in networks. *Phys Rev E Stat Nonlin Soft Matter Phys* 69, 026113. doi: 10.1103/PhysRevE.69.026113.

Pedregosa, F., Varoquaux, G., Gramfort, A., Michel, V., Thirion, B., Grisel, O., et al. (2011). Scikit-learn: Machine Learning in Python. *J. Mach. Learn. Res.*12, null (2/1/2011), 2825–2830.

Rand, W.M. (1971). Objective Criteria for the Evaluation of Clustering Methods. *J. Am. Stat. Assoc.* 66, 846-850. doi: 10.1080/01621459.1971.10482356.

van den Heuvel, M.P., and Sporns, O. (2011). Rich-club organization of the human connectome. *J. Neurosci.* 31, 15775-86. doi: 10.1523/JNEUROSCI.3539-11.2011.

Vinh, N., Epps, J., and Bailey, J. (2009). "Information theoretic measures for clusterings comparison: is a correction for chance necessary?", in.: ACM).

Watts, D.J., and Strogatz, S.H. (1998). Collective dynamics of 'small-world' networks. *Nature* 393, 440-2. doi: 10.1038/30918.

Wu, W.L. (2011). "Study of k-means and k-medoids algorithms in clustering analysis", in.: ProQuest Dissertations Publishing).

Zhu, Y., Wang, D., Liu, Z., and Li, Y. (2018). Aberrant topographical organization in default-mode network in first-episode remitted geriatric depression: a graph-theoretical analysis. *Int. Psychogeriatr.* 30, 619-628. doi: 10.1017/S1041610218000054.

**Supplementary Table 1. Modularity**

| Module Number | Member Regions (LH) | Member Regions (RH) |
| --- | --- | --- |
| module 1 | SFG_L_7_1, SFG_L_7_4,  MFG_L_7_1, MFG_L_7_2,  MFG_L_7_3, MFG_L_7_4,  MFG_L_7_6, MFG_L_7_7,  IFG_L_6_1, IFG_L_6_2,  IFG_L_6_3, IFG_L_6_4,  IFG_L_6_6, OrG_L_6_2,  OrG_L_6_6, ITG_L_7_1,  ITG_L_7_2, ITG_L_7_5,  ITG_L_7_6, ITG_L_7_7,  SPL_L_5_1, SPL_L_5_2,  SPL_L_5_5, IPL_L_6_2,  IPL_L_6_3, IPL_L_6_4,  Pcun_L_4_2, sOcG_L_2_2 | SFG_R_7_4, MFG_R_7_1,  MFG_R_7_2, MFG_R_7_3,  MFG_R_7_4, MFG_R_7_5,  MFG_R_7_6, MFG_R_7_7,  IFG_R_6_1, IFG_R_6_2,  OrG_R_6_2, ITG_R_7_2,  ITG_R_7_5, ITG_R_7_6,  ITG_R_7_7, SPL_R_5_1,  SPL_R_5_2, SPL_R_5_5,  IPL_R_6_2, IPL_R_6_3,  Pcun_R_4_2, sOcG_R_2_2 |
| module 2 | SFG_L_7_5, IFG_L_6_5,  STG_L_6_1, STG_L_6_2,  STG_L_6_3, STG_L_6_4,  STG_L_6_5, STG_L_6_6,  MTG_L_4_3, PhG_L_6_5,  pSTS_L_2_1, pSTS_L_2_2,  IPL_L_6_6, INS_L_6_1,  INS_L_6_2, INS_L_6_3,  INS_L_6_4, INS_L_6_5,  INS_L_6_6, CG_L_7_2,  CG_L_7_5, CG_L_7_6 | SFG_R_7_1, SFG_R_7_5,  IFG_R_6_3, IFG_R_6_4,  IFG_R_6_5, IFG_R_6_6,  OrG_R_6_6, PCL_R_2_1,  STG_R_6_1, STG_R_6_2,  STG_R_6_3, STG_R_6_4,  STG_R_6_5, STG_R_6_6,  MTG_R_4_3, pSTS_R_2_1,  pSTS_R_2_2, IPL_R_6_4,  IPL_R_6_6, INS_R_6_1,  INS_R_6_2, INS_R_6_3,  INS_R_6_4, INS_R_6_5,  INS_R_6_6, CG_R_7_3,  CG_R_7_5, CG_R_7_6 |
| module 3 | SFG_L_7_2, SFG_L_7_3,  SFG_L_7_6, SFG_L_7_7,  MFG_L_7_5, OrG_L_6_1,  OrG_L_6_3, OrG_L_6_4,  OrG_L_6_5, MTG_L_4_1,  MTG_L_4_2, MTG_L_4_4,  ITG_L_7_3, ITG_L_7_4,  IPL_L_6_1, IPL_L_6_5,  Pcun_L_4_1, Pcun_L_4_3,  Pcun_L_4_4, CG_L_7_1,  CG_L_7_3, CG_L_7_4,  CG_L_7_7, Cun_L_5_5 | SFG_R_7_2, SFG_R_7_3,  SFG_R_7_6, SFG_R_7_7,  OrG_R_6_1, OrG_R_6_3,  OrG_R_6_4, OrG_R_6_5,  MTG_R_4_1, MTG_R_4_2,  MTG_R_4_4, ITG_R_7_1,  ITG_R_7_3, ITG_R_7_4,  IPL_R_6_1, IPL_R_6_5,  Pcun_R_4_1, Pcun_R_4_3,  Pcun_R_4_4, CG_R_7_1,  CG_R_7_2, CG_R_7_4,  CG_R_7_7, Cun_R_5_5 |
| module 4 | FuG_L_3_1, PhG_L_6_1,  PhG_L_6_2, PhG_L_6_3,  PhG_L_6_4, PhG_L_6_6,  Amyg_L_2_1, Amyg_L_2_2,  Hipp_L_2_1, Hipp_L_2_2 | FuG_R_3_1, PhG_R_6_1,  PhG_R_6_2, PhG_R_6_3,  PhG_R_6_4, PhG_R_6_5,  PhG_R_6_6, Amyg_R_2_1,  Amyg_R_2_2, Hipp_R_2_1,  Hipp_R_2_2 |
| module 5 | FuG_L_3_2, FuG_L_3_3,  Cun_L_5_1, Cun_L_5_2,  Cun_L_5_3, Cun_L_5_4,  OcG_L_4_1, OcG_L_4_2,  OcG_L_4_3, OcG_L_4_4,  sOcG_L_2_1 | FuG_R_3_2, FuG_R_3_3,  Cun_R_5_1, Cun_R_5_2,  Cun_R_5_3, Cun_R_5_4,  OcG_R_4_1, OcG_R_4_2,  OcG_R_4_3, OcG_R_4_4,  sOcG_R_2_1 |
| module 6 | PrG_L_6_1, PrG_L_6_2,  PrG_L_6_3, PrG_L_6_4,  PrG_L_6_5, PrG_L_6_6,  PCL_L_2_1, PCL_L_2_2,  SPL_L_5_3, SPL_L_5_4,  PoG_L_4_1, PoG_L_4_2,  PoG_L_4_3, PoG_L_4_4, | PrG_R_6_1, PrG_R_6_2,  PrG_R_6_3, PrG_R_6_4,  PrG_R_6_5, PrG_R_6_6,  PCL_R_2_2, SPL_R_5_3,  SPL_R_5_4, PoG_R_4_1,  PoG_R_4_2, PoG_R_4_3, PoG_R_4_4, |
| module 7 | Str_L_6_1, Str_L_6_2,  Str_L_6_3, Str_L_6_4,  Str_L_6_5, Str_L_6_6,  Tha_L_8_1, Tha_L_8_2,  Tha_L_8_3, Tha_L_8_4,  Tha_L_8_5, Tha_L_8_6,  Tha_L_8_7, Tha_L_8_8 | Str_R_6_1, Str_R_6_2,  Str_R_6_3, Str_R_6_4,  Str_R_6_5, Str_R_6_6,  Tha_R_8_1, Tha_R_8_2  Tha_R_8_3, Tha_R_8_4,  Tha_R_8_5, Tha_R_8_6  Tha_R_8_7, Tha_R_8_8 |

**Supplementary Figures and Figure legends**

**Supplementary Figure1.** The analyses of the optimal number of clusters for the hierarchical-clustering solution. The silhouette coefficient, calculated for clusters 2 to 10 **(A)**. The Calinski-Harabasz index, calculated for clusters 2 to 10 **(B)**. The maximum achieved index values above indicated the best clustering of the data. The Davies–Bouldin index, calculated for clusters 2 to 10. A lower value will mean that the clustering is better**(C)**. The dendrogram for hierarchical clustering. The y-axis represents the distance between clusters. Various colors represent the two-cluster solution chosen **(D)**.

**
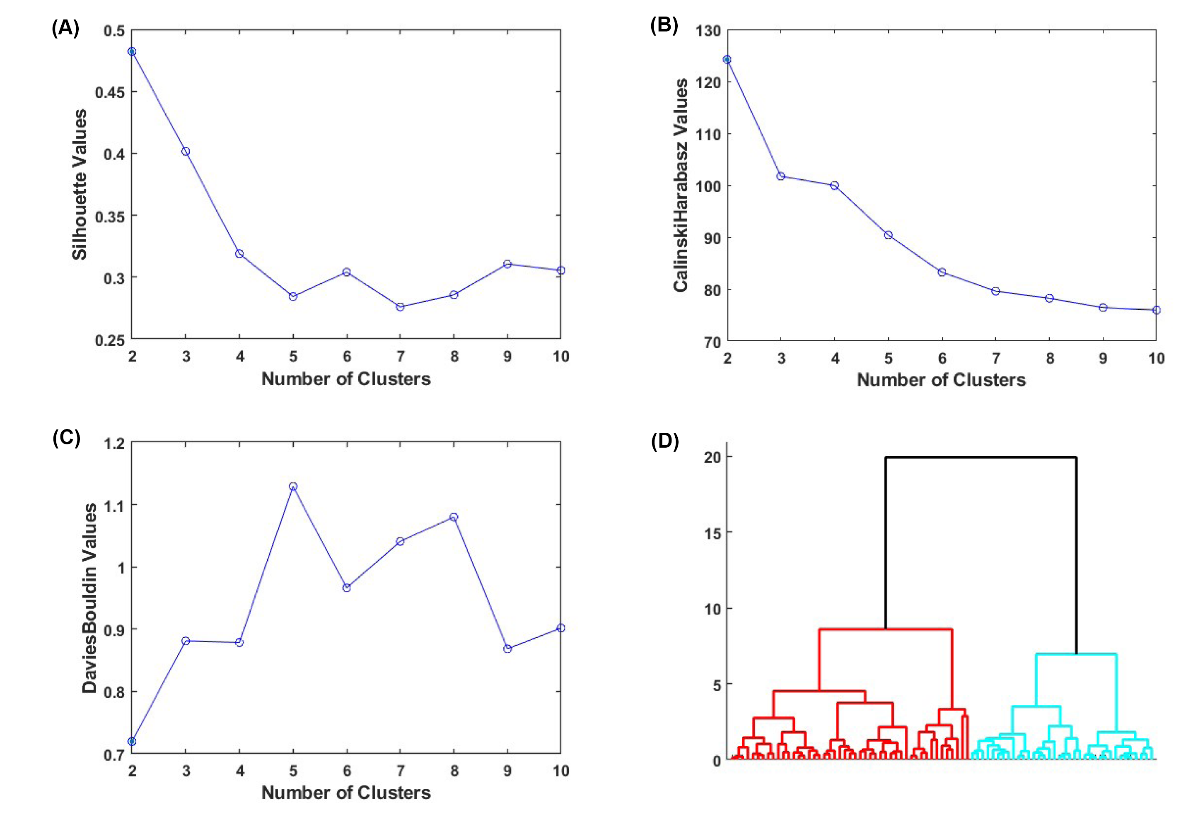
**

**Supplementary Figure2.** The analyses of the optimal number of clusters for the k-medoids solution. The silhouette coefficient, calculated for clusters 2 to 10 **(A)**. The Calinski-Harabasz index, calculated for clusters 2 to 10 **(B)**. The maximum achieved index values above indicated the best clustering of the data. The Davies–Bouldin index, calculated for clusters 2 to 10. A lower value will mean that the clustering is better **(C)**. The dendrogram for k-medoids. The y-axis represents the distance between clusters. Various colors represent the two-cluster solution chosen **(D)**.

**
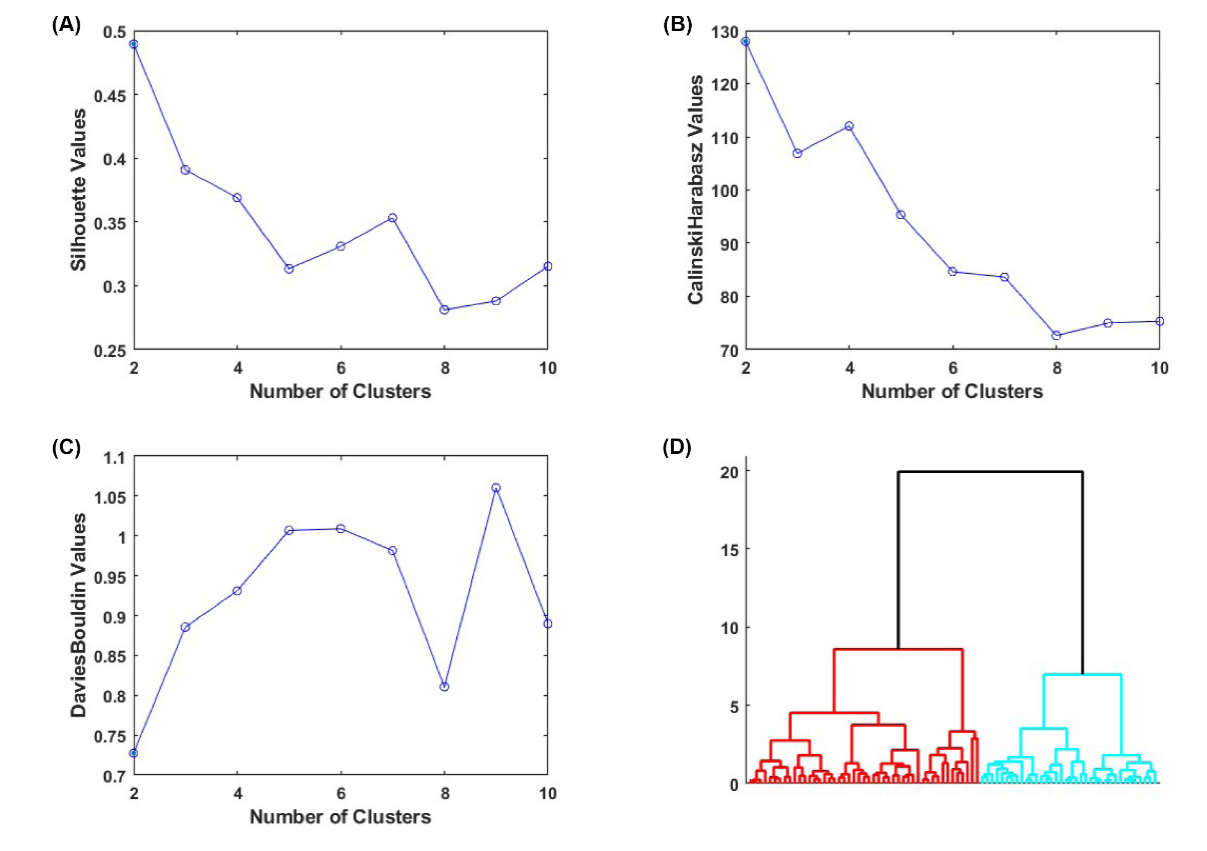
**

**Supplementary Figure3.** The analyses of the optimal number of clusters for the fuzzy C-means solution. The silhouette coefficient, calculated for clusters 2 to 10 **(A)**. The Calinski-Harabasz index, calculated for clusters 2 to 10 **(B)**. The maximum achieved index values above indicated the best clustering of the data. The Davies–Bouldin index, calculated for clusters 2 to 10. A lower value will mean that the clustering is better **(C)**. The dendrogram for fuzzy C-means. The y-axis represents the distance between clusters. Various colors represent the two-cluster solution chosen **(D)**.

**
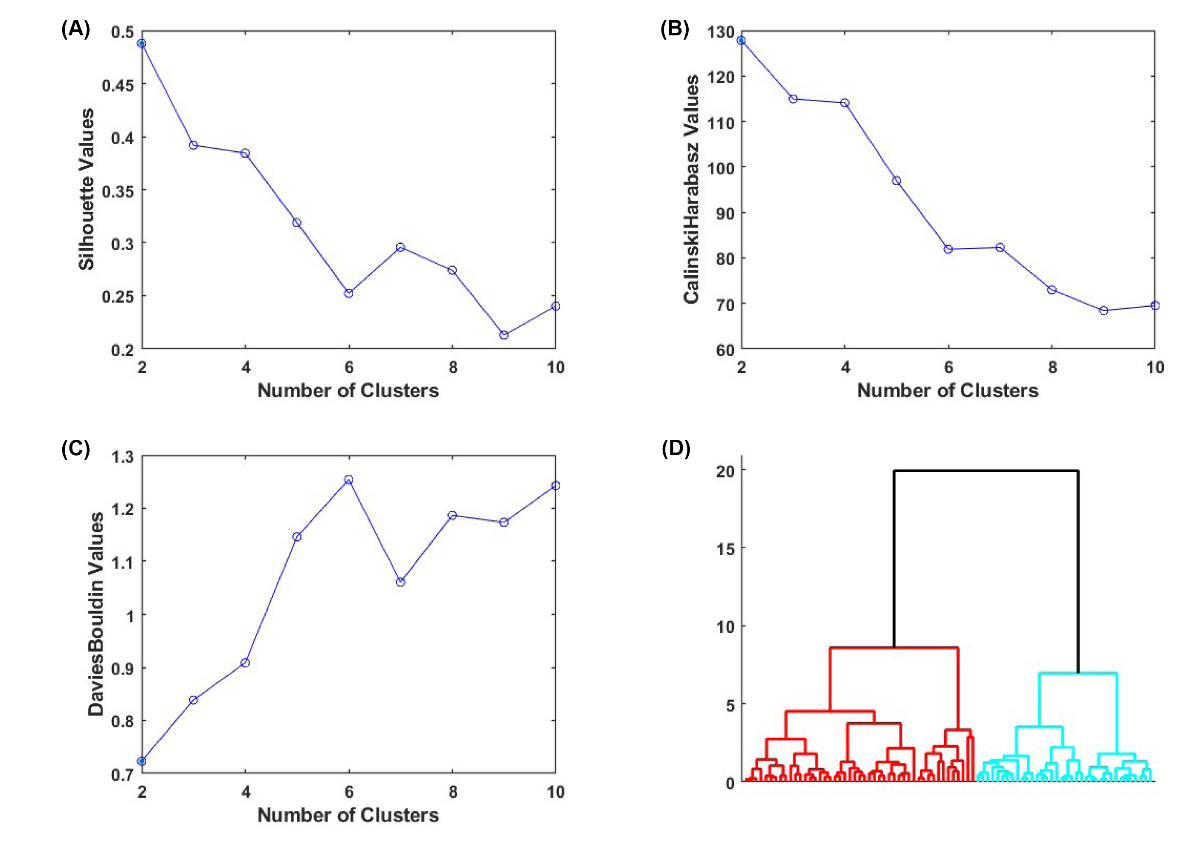
**

**SupplementaryFigure4.** Group effect in small-worldness of the functional connectome. The bar and error bar represent the mean values and standard error of these parameters of each group after removing the effects of age and gender, respectively. The stars indicate significant group effect (*P*<0.05/7≈0.0071).


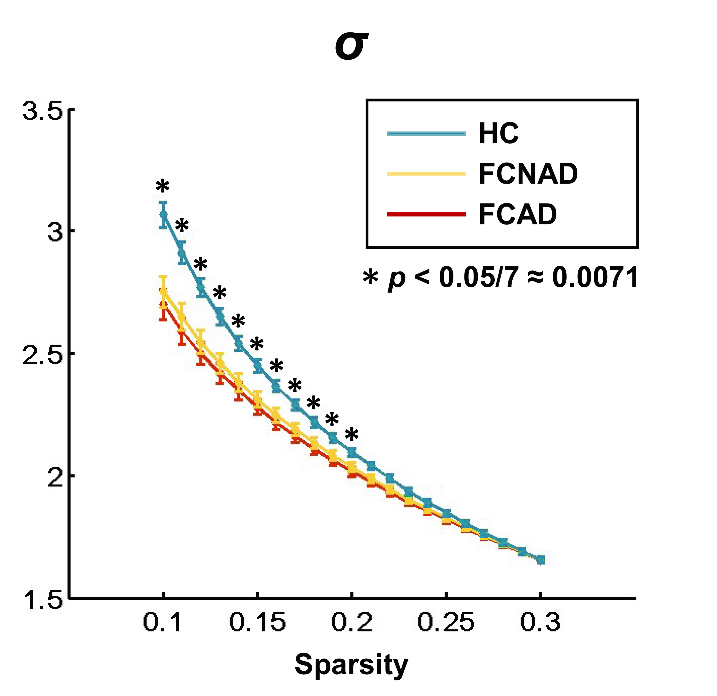


Abbreviations: *σ*, small-worldness; FCAD, functional constipation associated with anxiety/depressive status; FCNAD, functional constipation without anxiety/depressive status; HC, healthy controls.

**SupplementaryFigure5.** Group effects in global network metrics in FCAD, FCNAD and HC groups by using the “Willard” atlas. Among these three groups, there was significant group effect in *γ* and *σ* (*P*<0.05/7≈0.0071).


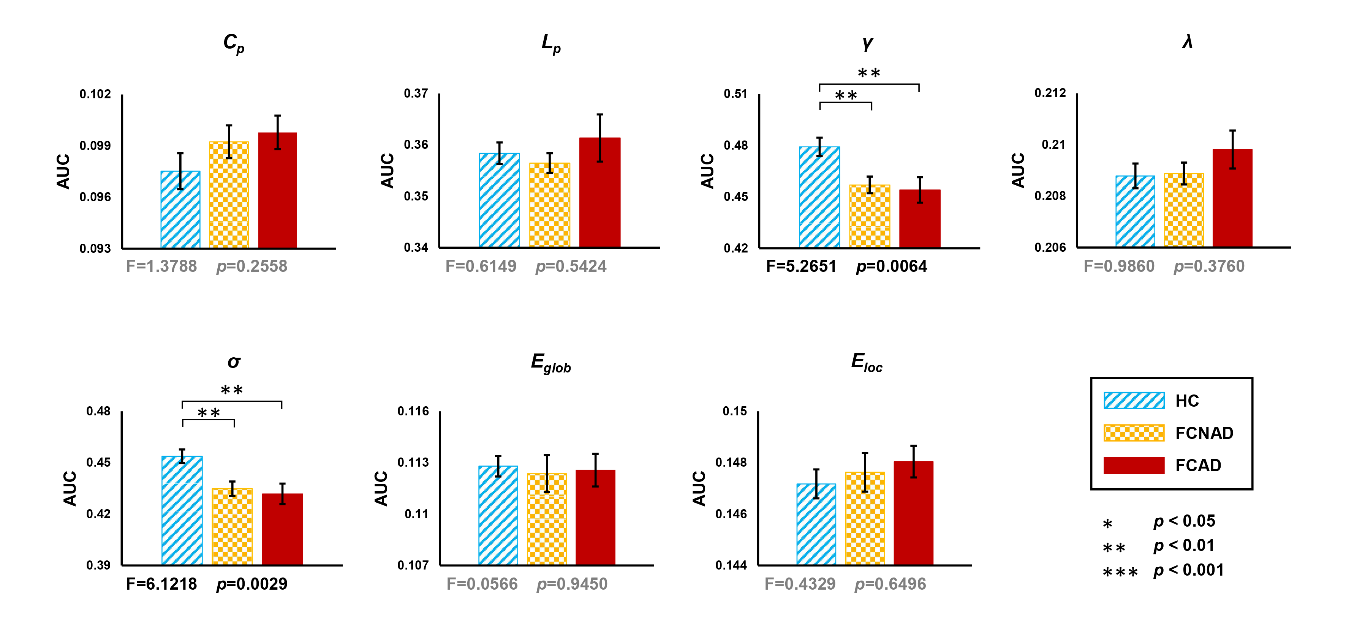


Abbreviations:$C_{p}$, clustering coefficient; $L_{p}$, shortest path length; *γ*, normalized clustering coefficient; *λ*, normalized shortest path length; *σ*, small-worldness; $E_{glob}$, global efficiency; $E_{loc}$, local efficiency. FCAD, functional constipation associated with anxiety/depressive status; FCNAD, functional constipation without anxiety/depressive status; HC, healthy controls.

**SupplementaryFigure6.** Group effects in global network metrics in FCAD, FCNAD and HC groups by using the Automated Anatomically Labeled (AAL) template. Among these three groups, there was significant group effect in *σ* (*P*<0.05/7≈0.0071).


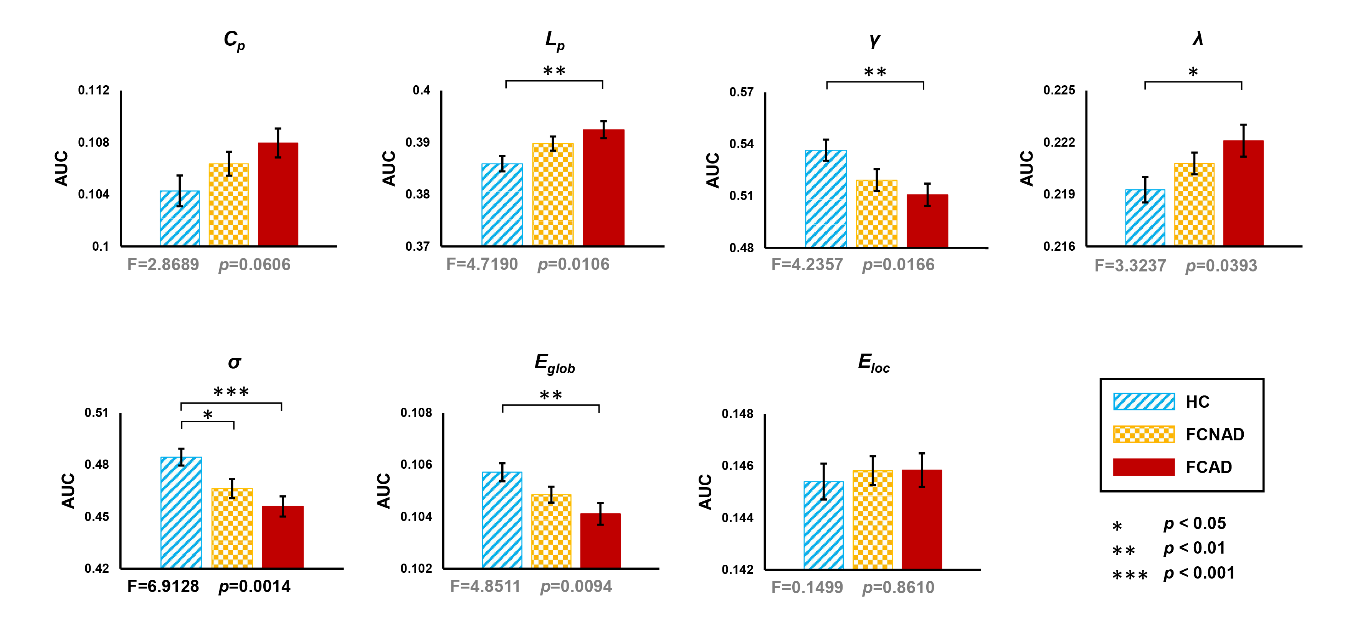


Abbreviations:$C_{p}$, clustering coefficient; $L_{p}$, shortest path length; *γ*, normalized clustering coefficient; *λ*, normalized shortest path length; *σ*, small-worldness; $E_{glob}$, global efficiency; $E_{loc}$, local efficiency. FCAD, functional constipation associated with anxiety/depressive status; FCNAD, functional constipation without anxiety/depressive status; HC, healthy controls.
